# The Prevalence of C-Section Delivery and Its Associated Factors Among Saudi Women Attending Different Clinics of King Khalid University Hospital

**DOI:** 10.7759/cureus.12774

**Published:** 2021-01-18

**Authors:** Hanan A Alabdullah, Lina Ismael, Lina A Alshehri, Sadeem Alqahtani, Munerah Alomari, Asma Alammar, Shaik Shaffi Ahamed

**Affiliations:** 1 Family & Community Medicine, College of Medicine, King Saud University, Riyadh, SAU

**Keywords:** cesarean section (cs), prevalence, saudi women, malpresentation

## Abstract

Objective

Cesarean section (C-section) is one of the most common surgical procedures worldwide that may be performed to deliver one or more newborns. The objective of our study was to determine the prevalence of C-section delivery among Saudi women attending different clinics of King Khalid University Hospital (KKUH) who were pregnant, previously pregnant, and had delivered.

Methods

A quantitative observational cross-sectional study using a self-administered questionnaire that has been handed to the participants after explaining the purpose of the study. A total of 524 pregnant and non-pregnant women were enrolled in the study randomly collected from all female clinics of KKUH. The study sample were given a self-administered questionnaire. Data were analyzed using the Statistical Package for Social Sciences (SPSS), version 21 (IBM SPSS Statistics, Armonk, NY) to analyze the data.

Results

Of the 524 study participants, 32.6% underwent C-section. There was a statistical significance in women aged 23 years old, as well as teachers, in relation to undergoing C-section (p-values = 0.0001 and 0.044, respectively). We concluded that malpresentation is the most common medical indication, with an overall percentage of 25%. There was no evident statistical association between body mass index (BMI), the highest level of education, marital status, Income status, mode of delivery, and the number of normal births or stillbirths.

Conclusion

It was found that the prevalence of Saudi women attending KKUH who underwent C-section was 32.6%. Medical indications to undergo C-sections, in comparison to the non-medical ones, were higher. Malpresentation was the most common medical indication, with an overall percentage of 25%.

## Introduction

Pregnancy is when a woman carries a developing embryo in her womb [1]. There are two modes of delivery, one of them is vaginal delivery and the less common mode is the cesarean section (C-section). C-section is one of the most common surgical procedures worldwide that may be performed to deliver one or more newborns [2-3]. C-sections performed appropriately and following appropriate medical indications are potentially life-saving procedures. There is a substantial increase in women who are undergoing C-section without any medical indication, and this may be the reason behind the global trend towards higher rates of C-section [4]. During the past several decades, cesarean deliveries in both developed and developing countries have reached an alarmingly high rate. Based on a study conducted by the World Health Organization (WHO) in 2008, it was estimated that approximately 18.5 million C-section procedures were performed yearly with 69 countries having C-section rates above 15% [3]. In Saudi Arabia, the C-section rate accounts for around 10% of all births, reaching 20% in tertiary centers. The observed increase in cesarean birth has been attributed to many factors, including the first birth, in particular, and advanced maternal age. Other factors include the obstetrician's characteristics and care practices [5].

Assessment of the current situation regarding the increasing numbers of C-section in the Kingdom of Saudi Arabia (KSA) consequently increases the number of complications for the mother and child if it is not due to a life-threatening medical indication, not to speak of its economic impact on the families. Although the surgery might be preferred in cases of a medical indication, it still has its surgical risks that may jeopardize both the mother and child’s health [2]. Recently, C-sections have been performed to satisfy women’s desire; therefore, cesarean delivery on maternal request (CDMR) was added to the known common C-section indications [6]. Our study aims to contribute to the process of raising awareness regarding the perceived idea that C-section is safer than normal delivery by pinpointing the factors that influence women’s choice of delivery.

There are a limited number of studies conducted in Saudi Arabia and, more specifically, in Riyadh that studied or quantified the associated factors that influence women’s choice of undergoing C-section delivery. In our research, our objective is to determine the prevalence of C-section delivery among Saudi women attending different clinics of King Khalid University Hospital (KKUH) who were pregnant, previously pregnant, and those women who had delivered.

## Materials and methods

This study was approved by the Institutional Review Board at King Saud University (KSU) College of Medicine. Oral or written consent was obtained from study subjects prior to administering a questionnaire. Names and other identifying information from the study subjects were not requested during data collection. Confidentiality of data was maintained, and the data was used only for this research study.

The study design was an analytical quantitative observational cross-sectional study from December 2017 to January 2018. We targeted Saudi females who were pregnant, had previously been pregnant, and had delivered and who attended different clinics at KKUH, which included medicine, surgery, obstetrics and gynecology (OB/GYN), dermatology, phlebotomy, and lastly, the primary care clinic.

Sample size calculation was done using an online tool (http://www.raosoft.com/). Using a 25% prevalence, a 4% margin of error, and a 95% confidence level, the calculated sample size was 441. However, the authors aimed to increase the number of participants to 540 to compensate for missing data and non-response. However, we were only able to collect 524 responses.

Convenient sampling based on different times from different female clinics was used as the sampling method. All Saudi women from the age of 16 years old and above who were previously or currently married were included. The questionnaire had 24 questions which were prepared in English and Arabic based on a literature search to answer our objectives by the mutual efforts of all authors (see Appendix). The questionnaire was developed by the authors after a thorough review of the literature and was translated into Arabic. Questions were closed-ended, open-ended, and some had binary answers (yes/no). Data collectors were trained in the use of the questionnaire. Participants were given a self-administered questionnaire.

Data were analyzed using the Statistical Package for Social Sciences (SPSS), version 21 (IBM SPSS Statistics, Armonk, NY). Descriptive statistics that were used included the mean, standard deviation, frequencies, and percentages. Chi-square and student t-test for independent samples were used for bivariate analysis. A p-value of < 0.05 and 95% confidence intervals were used to report the statistical significance and precision of results. 

## Results

Of the 524 study participants, the ages varied widely, but the frequency was the highest in women aged > 50 (20.4%) and the lowest in the ones aged < 20 (1.1%). Housewives held the highest rate amongst other occupations, teachers came in second place, and there was a variable degree in other occupations. In regard to marital status, (88.5%) of the women were married and the rest were either divorced or widows. Having an income status of (5000 - 10,000 Saudi Riyal (SR)) was represented in approximately 50% of our sample size, and only 5% had ≥ 20,000 SR. An overweight, Class I obesity, and normal weight body mass index (BMI) were ranked highest to lowest with percentages of 27.1%, 21.4%, and 18.1%, respectively. Many of the women in our study had a bachelor's degree, which represented as many as 44% of the total sample. Sociodemographic variables are shown in Table 1. However, of all of the participants, 32.6% of women underwent C-section and 67.4% of women had a normal delivery.

**Table 1:** Characteristics of the Study Subjects BMI: body mass index; PhD: Doctor of Philosophy

Social demographic	N (%)
Age	
< 20	6 (1.2)
21 - 25	36 (6.9)
26 - 30	81 (15.6)
31 - 35	65 (12.5)
36 - 40	99 (19)
41 - 45	48 (9.2)
46 - 50	78 (15)
> 50	107 (20.6)
Occupation	
Teacher	92 (17.7)
Student	33 (6.3)
Housewife	308 (59.1)
Other	88 (16.9)
Marital Status	
Married	464 (88.6)
Divorced	28 (5.3)
Widow	32 (6.1)
Income Status	
< 5,000	148 (29)
5,000 - 10,000	232 (45.5)
10,000 - 20,000	104 (20.4)
≥ 20,000	26 (5.1)
BMI	
Normal body weight	95 (22.4)
Overweight	142 (33.6)
Class I obesity	112 (26.4)
Class II obesity	43 (10.1)
Class III obesity	32 (7.5)
Level of education	
Primary school or less	101 (19.3)
Intermediate	44 (8.4)
High school	123 (23.5)
University (undergrad)	230 (43.9)
Masters, PhD	26 (4.9)

There was no association between BMI, the highest level of education, marital status, income status, and the mode of delivery. On the other hand, a noteworthy association between categories of occupation and c-section was found. This might indicate that poor physical activity is related to C-sections. Also, women who were older in age had a significant association between their ages and C-section delivery (Table 2). 

**Table 2:** Comparison of Qualitative Study Variables in Relation to Mode of Delivery in Saudi Women BMI: body mass index; PhD: Doctor of Philosophy

Variables	Mode of delivery (%)		Χ^2^ value	P-value
	Cesarean delivery	Normal delivery		
Age				
< 20	1 (16.7)	5 (83.3)	29.162	< 0.0001
21 - 25	2 (5.6)	34 (94.4)		
26 - 30	21 (25.9)	60 (74.1)		
31 - 35	23 (35.4)	42 (64.6)		
36 - 40	40 (40.4)	59 (59.6)		
41 - 45	23 (47.9)	25 (52.1)		
46 - 50	33 (42.3)	45 (57.7)		
> 50	26 (24.3)	81 (75.7)		
BMI				
Normal body weight	28 (29.5)	67 (70.5)	4.346	0.361
Overweight	42 (29.6)	100 (70.4)		
Class I obesity	39 (34.8)	73 (65.2)		
Class II obesity	13 (30.2)	30 (69.8)		
Class III obesity	15 (46.9)	17 (53.1)		
Highest level of education				
Primary school or less	31 (30.7)	70 (69.3)	4.138	0.388
Intermediate	20 (45.5)	24 (54.5)		
High school	42 (34.1)	81 (65.9)		
University (undergraduate)	70 (30.4)	160 (69.6)		
Masters, PhD	8 (30.8)	18 (69.2)		
Occupation				
Teacher	38 (41.3)	54 (58.7)	8.116	0.044
Student	5 (15.2)	28 (84.8)		
Housewife	102 (33.1)	206 (66.9)		
Other	26 (29.5)	62 (70.5)		
Marital status				
Married	149 (32.1)	315 (67.9)	1.417	0.496
Divorced	12 (42.9)	16 (57.1)		
Widow	10 (31.3)	22 (68.8)		
Income status				
< 5,000	47 (31.8)	101 (68.2)	1.654	0.647
5,000 - 10,000	71 (30.6)	161 (69.4)		
10,000 - 20,000	39 (37.5)	65 (62.5)		
≥ 20,000	9 (34.6)	17 (65.4)		

Further tests for significance were done. A statistical significance was found between the number of children and the method of delivery. There was also statistical significance in the age when delivering the first child, while there was no statistical significance in the number of normal births or stillbirths (Table 3). 

**Table 3:** Comparison of Mean Values of Quantitative Study Variables in Relation to Mode of Delivery in Saudi Women CI: confidence interval

Variables	Mode of delivery		Mean difference	T-value	P-value	95% CI of the difference of mean
	Cesarean delivery	Normal delivery				
Number of children	4.58	4.01	0.576	1.979	0.048	0.004 - 1.148
Number of live births	4.16	3.74	0.426	1.642	0.101	-0.084 - 0.935
Number of stillbirths	0.69	0.61	0.078	0.677	0.499	-0.149 - 0.305
Age when delivering your first child?	23.01	19.80	3.215	4.768	< 0.0001	1.890 - 4.540

Both groups of women who underwent vaginal delivery (64.4%) and C-section (35.6%) preferred the doctor to be the one who makes the decision regarding the mode of delivery. They believed that women in our society preferred vaginal delivery to a C-section. Also, the answers indicated that vaginal delivery had fewer complications for the mother. Both groups of women who underwent C-section and vaginal delivery sometimes encountered women who preferred to deliver by C-section. They also believed that our community is moderately aware of the C-section complications with a percentage of 30.5% and 69.5%, respectively (Table 4).

**Table 4:** Association of the Perceptual Study Variables with the Mode of Delivery

Variables	Mode of delivery (%)			Χ^2^ value	p-value
	Cesarean delivery (C-section)		Normal delivery		
Who has the right to choose the mode of delivery?					
Family members	7 (50)	7 (50)		6.175	0.103
Doctor	104 (35.6)	188 (64.4)			
You	60 (27.5)	158 (72.5)			
On a scale from 1 to 5, how much does your answer to the previous question affect your choice in C-section?					
1	17 (41.5)	24 (58.5)		2.855	0.582
2	4 (36.4)	7 (63.6)			
3	18 (32.7)	37 (67.3)			
4	24 (33.3)	48 (66.7)			
5	89 (29.3)	215 (70.7)			
In your community, what do you think women prefer?					
Vaginal	141 (31.9)	301 (68.1)		1.463	0.226
C-section	30 (36.6)	52 (63.4)			
From your point of view, which method has fewer complications (mother)?					
Vaginal	141 (31.9)	301 (68.1)		0.691	0.406
C-section	30 (36.6)	52 (63.4)			
From your point of view, which method has fewer complications (infant)?					
Vaginal	113 (29.6)	269 (70.4)		5.520	0.019
C-section	57 (40.0)	84 (59.6)			
In your community, how often you encounter women who prefer to deliver by C-section?					
Not at all	16 (40)	24 (60.0)		5.502	0.138
Rarely ever	51 (34.5)	97 (65.5)			
Sometimes	77 (34.5)	146 (65.5)			
More frequently	27 (23.9)	86 (76.1)			
In your community, how would you assess the awareness about the complication?					
Not aware	19 (24.4)	59 (75.6)		5.168	0.159
Very little	43 (34.7)	81 (65.3)			
Moderately aware	57 (30.5)	130 (69.5)			
Highly aware	52 (38.5)	83 (61.5)			

We concluded that malpresentation was the most common medical indication with an overall percentage of 25%, which is a quarter of the whole sample we distributed the data amongst. Pelvic organ prolapse, which has the least percentage of only 0.6%, indicates that it is not an important indication (Table 5).

**Table 5:** Distribution of Medical Indications of Undergoing C-section C-section: cesarean section

Medical indications of undergoing C-section frequencies	N (%)
Failure to progress in labor (dystocia)	28 (17.5)
Malpresentations	40 (25)
Acute fetal distress	4 (2.5)
Preterm births	9 (5.6)
Placental abruption	4 (2.5)
Placenta previa	2 (1.3)
Failed induction of labor	3 (1.9)
Hypertensive disorders	15 (9.4)
Prolonged pregnancy	3 (1.9)
Pelvic organ prolapse	1 (0.6)
Birth defect	2 (1.3)
Nuchal cord	3 (1.9)
Ectopic pregnancy	3 (1.9)
Gestational diabetes	8 (5)
Weak fetal pulse	9 (5.6)
Hemorrhage	2 (1.3)
Other	24 (15)
Total	160 (100)

## Discussion

In 1998, the prevalence of Australian women who gave birth by C-section was 21%, which was increased to 30.9% in 2007 [7]. In Egypt, the overall rate of delivery by C-section was 47.25%, which is higher than other rates quoted across the globe, both in developed and developing countries [8]. C-section reached about 10% of total births in Saudi Arabia and approximately reaching 20% in tertiary centers. The overall C-section rate increased from 10.6% in 1997 to 19.1% in 2006, corresponding to an 80.2% increase [5]. Over the last 20 years, a steady increase in the C-section delivery rate was observed at King Abdulaziz Medical City, Riyadh, Saudi Arabia [9]. C-section delivery rate advanced from 8% to 21% between the years 1993 and 2013. Our study shows that out of 524 study participants, 32.6% of women underwent a C-section. The occupation was significantly associated with C-section when compared with other variables. Knowing which occupation has higher rates of C-section may suggest other associated factors, such as physical activity, as some occupations may involve more physical activity than others. The rates of C-section vary from nation to nation. Saudi Arabia can be considered to have a bit higher rates of C-sections. The rates are acceptable in the United States, for example. One study showed that 11.6% had a non-indicated C-section, while 14.2% were indicated, and the rest underwent normal vaginal delivery [10]. The countries of South and Southeast Asia have different rates of C-section ranging from 1.51% to 58.54% [11]. The Maldives had the highest C-section rates (31.78%) based on institutional and non-institutional births, while Bangladesh had the highest rates (58.54%) based on institutional births. In Pakistan, one study reported a prevalence of 44.8%, of which 41.4% were elective C-sections [12]. In the same study, those who were nulliparous and done C-section represented 30%, this can be a warning sign since that currently the most common indication in was previous C-sections, accounting for 30.9%. On the other hand, our results suggest that the rate of C-section in Saudi Arabia are to be reviewed and to educate both patients and the treating doctors to include counseling about normal vaginal delivery for all elective C-section patients, if possible. 

The imitations of our study were that our study design is cross-sectional and, therefore, we are only able to suggest associations rather than causal relationships. The sample subjects were from one public hospital (KKUH) exclusively, which limits the generalizability of our findings. The reliability of the questionnaire should be reassessed. These limitations could be overcome by repeating the same study as a cohort (prospective or retrospective). Also, widening the sample subjects to include participants from both private and public hospitals will lead to more confident generalization. Further studies should be conducted to investigate the high prevalence of malpresentation. Since we concluded that in the perception of our study subjects, the majority of our community were unaware of C-section complications, awareness campaigns should be conducted. Our study aimed to identify the prevalence of C-sections and understand the associated factors that influence a woman's choice to undergo that procedure. Filling the gap of knowledge about this issue should provide the basis for further research in order to solve the problem of increasing C-sections. A reduction in rates is necessary since it has been reported that vaginal delivery is safer than C-section delivery concerning maternal mortality. Reducing the rate of an unnecessary C-section will lower its associated complications. Our future direction is to investigate the prevalence of C-section in private hospitals in Riyadh and compare them with our results at KKUH.

## Conclusions

We found that the prevalence of Saudi women attending KKUH who underwent a C-section was 32.6%. Medical indications to undergo C-sections in comparison to the non-medical ones were higher. Malpresentation is the most common medical indication, with an overall percentage of 25%. Further studies should be conducted to investigate the high prevalence of malpresentation, as well as widening the spectrum of Saudi females included, such as those from private hospitals. We also recommend that awareness campaigns should be conducted since the majority of our community were unaware of C-section complications.

## Appendices

Prevalence of C-section delivery and its associated factors among Saudi women attending different clinics of King Khalid University Hospital (KKUH)

We are students from the College of Medicine, King Saud University studying the prevalence of cesarean section (C-section) and to understand the associated factors that influence a woman's choice to undergo it among pregnant and non-pregnant (previously pregnant and delivered) Saudi women attending different clinics of King Khalid University Hospital (KKUH) between 2017-2018.

Your participation in this study will be kept confidential. The results of this research may be published; however, your identity will never be revealed.  

Personal information:

1) Age:                            years

2) Height:                       cm

3) Weight:                       kgs

4) Highest level of education:

                1. Primary school or less            ▢     

                2. Intermediate                           ▢

                3. High school                            ▢    

                4. University (undergrad)           ▢

                5. Masters, PHD                        ▢

5) Occupation:   

                1. Doctor                               ▢                      

                2. Banker                              ▢

                3. Nurse                                ▢                     

                4. Pharmacist                        ▢

                5. Teacher                            ▢                     

                6. Student                             ▢

                7. Housewife                         ▢                     

                8. Cashier                            ▢

                9. Other:                                ▢

6) Marital status:

                1. Single         ▢  

                2. Married       ▢  

                3. Divorced     ▢  

                4. Widow         ▢

7) Income status:

                 1. < 5000                 ▢              

                 2. 5,000 - 10,000    ▢  

                 3. 10,000 - 20,000  ▢

                 4. 20,000-50,000    ▢

                 5. > 50,000              ▢

Pregnancy:

8) Are you pregnant now? 

                 ▢ 1. Yes                             ▢  2. No (If no, go to question #9)      

                 ▢ 1.a Primigravida           ▢  1.b Multigravida   

Gestational Week:    ___________________

9) Number of live births:  ___________________

10) Number of stillbirths:   ____________________

11) Previous spontaneous abortion:

                ▢  1. Yes          ▢   2. No     

Reason:  ___________________

12) Previous abortion:       

                ▢ 1. Yes           ▢   2. No     

13) Number of children:   ____________________

14) Age of youngest child: ___________________

15) What was your age when delivering your first child? ____________________

Delivery:  

16) Have you ever have had a cesarean section?  

               ▢ 1. Yes                     ▢ 2. No (If no, go to question #17) 

Number of cesarean sections: ___________________

Where did you do it? 

               ▢ 1. Private hospital   ▢  2. Public hospital   

If C-section, ask why? ___________________

16-A) Medical indication: 

               ▢ 1. Yes            ▢  2. No (If no, go to the next question #16.B)

Specify the reason:

              1.   Failure to progress in labour (dystocia)       ▢

              2.  Malpresentations                                          ▢

              3.   Acute fetal distress                                       ▢

              4.   Preterm births                                               ▢

              5.   Placental abruption                                      ▢

              6.   Placenta praevia                                           ▢

              7.   Failed induction of labor                               ▢

              8.   Hypertensive disorders                                ▢

              9.   Prolonged pregnancy                                   ▢

             10.  Pelvic organ prolapse                                     ▢

             11.  Urinary incontinence                                       ▢

             12. Sexually transmitted disease                          ▢

             13.  Other: ________________

16-B) Electively on request C-section:  1. Yes  ▢     2. No   ▢

               1.  Family choice:                                          ▢

                   If yes please specify:  __________

               2. Society influence                                      ▢       

                    If yes please specify:  __________

               3. Tokophobia, fear of labor & childbirth    ▢

               4. Cosmetics:                                                 ▢

               5. To avoid the episiotomy/tear                   ▢

               6. Longer maternity leave                             ▢

               7. Other: ________________                      ▢

17) How many vaginal deliveries have you had?  ________________

18) Who has the right to choose the mode of delivery, in your opinion?    

                1. Mother                  ▢   

                2. Husband              ▢  

                3. Doctor                  ▢

                4. You                       ▢

19) On a scale from 1 to 5, how much does your answer to question #17 affect your choice?

                 1 ▢     2 ▢      3 ▢      4 ▢       5 ▢

20) In your community, what do you think women prefer:  

                 1. Vaginal delivery    ▢    2. Cesarean delivery   ▢

21) From your point of view, which method has less complication (for the mother):  

                  1. Vaginal delivery    ▢    2. Cesarean delivery ▢

22) From your point of view, which method has less complication(for the Baby): 

                   1. Vaginal delivery    ▢    2. Cesarean delivery ▢

23) In your community, how often you encounter women who prefer to deliver by cesarean section?

                   1 ▢     2 ▢      3 ▢      4 ▢       5 ▢

24) In your community, how would you assess the awareness about the complications of cesarean section?

                    1 ▢     2 ▢      3 ▢      4 ▢       5 ▢
